# Multi-trait analysis of pigmentation traits in relation to skin and ocular cancers: Relevance for evolutionary medicine

**DOI:** 10.1093/emph/eoag021

**Published:** 2026-09-10

**Authors:** Cristina L Abbatangelo, Brendan Newton, Lydia M Li, Esteban J Parra

**Affiliations:** Department of Anthropology, Faculty of Arts and Science, University of Toronto, Toronto, Ontario, Canada; Department of Anthropology, University of Toronto Mississauga, Mississauga, Ontario, Canada; Departmen of Biology, McMaster University, Hamilton, Ontario, Canada; Genetics and Genome Biology Program, The Hospital for Sick Children, Toronto, Ontario, Canada; Institute of Medical Science, Temerty Faculty of Medicine, University of Toronto, Toronto, Ontario, Canada; Department of Applied Pyschology and Human Development, University of Toronto, Toronto, Ontario, Canada; Department of Anthropology, University of Toronto Mississauga, Mississauga, Ontario, Canada

**Keywords:** pigmentation, skin cancer, ocular cancer, multi-trait analysis

## Abstract

**Background and objectives:**

Pigmentary traits (skin, hair, eye colour, tanning response) are epidemiologically linked to skin and ocular cancers, but the extent and nature of shared genetic architecture remain incompletely defined. In this study we explored to what extent pigmentation- and cancer-associated variants exhibit pleiotropy.

**Methodology:**

We analyzed genome-wide association studies summary statistics from European populations for skin colour; hair colour (blonde, light brown, red, dark brown, black); eye colour; tanning response; and four cancers: cutaneous malignant melanoma (CMM), basal cell carcinoma (BCC), squamous cell carcinoma (SCC), and uveal melanoma (UM). Genetic correlations were estimated with LD score regression. Multi-trait and pairwise pleiotropy were tested with Cross-Phenotype Association. We reviewed available colocalization data (COLOC framework) and post-association functional annotation/mapping (e.g. FUMA) to interpret shared signals.

**Results:**

Significant genetic correlations were observed between multiple pigmentary traits and each cancer. Multi-trait and pairwise analyses identified numerous shared regions, including canonical pigmentation loci (*MC1R, ASIP, TYR, BNC2, IRF4, OCA2/HERC2, SLC24A4, DCT, TYRP1*). Directions of effect indicated that lighter skin, hair and iris pigmentation and poor tanning were associated with increased risk across cancers. Colocalization data supported shared causal variants at several loci; for UM, variants driving light eye colour at *IRF4* and *HERC2* were linked to higher risk.

**Conclusions and implications:**

Skin and ocular melanomas illustrate evolutionary mismatch: variants favoured at high latitudes for lighter pigmentation now elevate cancer risk under modern UV exposure. Mapping shared architecture highlights biological pathways that may refine risk prediction, motivate functional studies, and inform prevention strategies.

**Lay summary:**

Using genome-wide data, we examined how genes shaping human pigmentation relate to risk of skin and eye melanomas. Multi-trait analyses revealed shared genetics: lighter skin, hair and eye colour, and poor tanning, increased cancer risk. Findings illustrate evolutionary mismatch and highlight pathways that guide prevention, risk prediction, and future research.

## INTRODUCTION

Eumelanin, the darker and more photoprotective form of melanin, acts as a natural sunscreen, absorbing and scattering ultraviolet (UV) radiation to limit DNA damage, preserve folate (a vitamin essential for embryonic development), and reduce the risk of UV-associated skin cancers [1]. The regulation of melanin type and distribution across skin, hair, and ocular tissues is increasingly well characterized at the genetic level [2–5].

Although melanin affords substantial protection, the evolution of dark pigmentation in the genus *Homo* was likely not driven primarily by cancer avoidance [6]. Most skin cancers, including basal cell carcinoma (BCC), squamous cell carcinoma (SCC), and cutaneous malignant melanoma (CMM), tend to develop after peak reproductive years and generally have low mortality [6–8]. Nevertheless, modern epidemiology shows higher incidence in fair-skinned individuals than in those with darker pigmentation [9].

Beyond skin pigmentation, other melanin-based traits such as freckling, tanning ability, red hair, and light-coloured eyes elevate risk [10–14]. Mendelian randomization analyses have strengthened the causal interpretation of these associations; for instance, darker natural hair colour has been reported as causally protective against BCC, SCC, and CMM [15]. Genes that have been associated with pigmentation phenotypes, including *SLC45A2, IRF4, OCA2, TYR, BNC2, SLC24A4, ASIP, SLC24A5, MITF*, and *MC1R*, have also been associated with increased susceptibility to skin cancers [11–13, 16]. The breadth of these associations across skin cancers has been systematically documented in large-scale genome-wide association studies (GWAS), with repositories such as the NHGRI-EBI GWAS Catalogue providing a consolidating reference for replicable pigmentation-cancer loci [17, 18]. Notably, *MC1R* variants linked to red hair and pale skin show strong associations with CMM, BCC, and SCC [19, 20].

UV radiation remains the primary driver of skin cancer development, with fair-skinned populations in high-UV environments experiencing the greatest burden. This is particularly evident in countries such as Australia, where the combination of intense UV exposure and high rates of sun-sensitive phenotypes has resulted in elevated incidence of CMM [7, 9]. In Canada, CMM incidence is also rising with southern and coastal provinces, especially Prince Edward Island and Nova Scotia, exhibiting the highest melanoma rates [21]. Emerging climate models project increased UV irradiance in several regions in coming decades [22], reinforcing the importance of understanding shared genetic pathways between pigmentation and skin cancer.

Although most work on pigmentation and cancer risk centres on skin, pigmentation is also critical in ocular tissues. Melanocytes in the uveal tract, choroid, ciliary body and iris can give rise to uveal melanoma (UM), a rare but consequential cancer in which nearly 50% of patients eventually develop metastases, typically to the liver [23]. Risk increases with age, lighter pigmentation (fair skin, light-coloured irises), cutaneous nevi, nevus of Ota, and family history of UM or cutaneous melanoma. Across Europe, UM incidence follows a latitudinal gradient, ranging from ~10 cases per million annually in Scandinavia to ~3 per million in Southern Europe [24]. Clinically, UM may present through vision disturbances, iris colour changes, or visual field loss [25].

To date, seven genome-wide significant loci have been associated with UM risk [26, 27], two of which have previously been associated with eye colour variation (rs12203592 and rs12913832) [28, 29]. The strongest signal maps to *IRF4*, a transcription factor involved in both melanogenesis and immune regulation [30, 31]; this variant follows and additive pattern for UM risk, with risk increasing with the number of alleles [26]. The second signal lies in *HERC2* and regulates *OCA2*, a key determinant of blue eye colour [32–34]; although quasi-recessive for eye colour itself [32, 35], its effects on skin and hair pigmentation, and now UM susceptibility, appear largely additive [26, 35]. A third locus maps to *CLPTM1L/TERT*, previously implicated in several cancers including CMM and lung cancer [36]. These three signals have been independently replicated, with four additional novel loci reported, bringing the total to seven [26]. Together, epidemiological and genetic evidence consistently link light eye colour to increased UM risk.

### The present study

This project investigates shared genetic architecture between pigmentation traits, skin, and ocular cancer susceptibility using multi-trait genomic methods. We leverage summary statistics from GWAS for pigmentation phenotypes, including skin colour, hair colour (blonde, light brown, red, dark brown, black), and tanning response, from the UK Biobank and the Canadian Partnership for Tomorrow’s Health (CanPath). These traits are analyzed alongside GWAS summary statistics for CMM, SCC, BCC, and, for the first time in a multi-trait framework, UM, following the public release of UM GWAS data in 2025 [26].

## METHODOLOGY

### Genome-wide datasets

This study utilizes de-identified publicly available processed GWAS summary statistics for both sexes curated primarily from the UK Biobank (UKB) [37, 38], with additional contributions from QSkin Sun and Health Study (QSkin) [39], eMERGE (dbGaP, study accession: phs000360.v3.p1), GERA (dbGaP, study accession: phs000674.v3.p3), Willis Eye Hospital (Philadelphia, PA, USA) [26], Penn Medicine Biobank [40] and the Canadian Partnership for Tomorrow’s Health [41].

The UKB includes >500 000 individuals (mean age 57) recruited in 2006–2010; all participants provided consent. To minimize population stratification, analyses were restricted to individuals of European descent. Phenotypes analyzed are listed in Table 1, along with sample sizes and study details. The summary statistics were accessed from the NHGRI-EBI GWAS Catalogue (https://www.ebi.ac.uk/gwas/) and GWAS Atlas (https://atlas.ctglab.nl/).

**Table 1:** Summary of phenotypes used in multi-trait analysis. Sample size (N), case and control sample size (N_case_ and N_control_ respectively), GWAS model, population ancestry and SNP heritability (h^2^_SNP_) for each set of summary statistics is provided.

Phenotype	Sample Size and GWAS Model	Cohort and Ancestry	Web Access	SNP-based Heritability (h ^2^ _SNP_ )
Blonde hair colour [42]	N_case_ = 43 319, N_control_ = 342 284, N = 385 603 (logistic)	UK Biobank (EUR)	https://atlas.ctglab.nl/traitDB/3495	h^2^_SNP=_0.299 (SE = 0.044)
Red hair colour [42]	N_case_ = 17 329, N_control_ = 368 274, N = 385 603 (logistic)	UK Biobank (EUR)	https://atlas.ctglab.nl/traitDB/3496	h^2^_SNP=_0.076 (SE = 0.069)
Light brown hair colour [42]	N_case_ = 155 612, N_control_ = 229 991, N = 385 603 (logistic)	UK Biobank (EUR)	https://atlas.ctglab.nl/traitDB/3497	h^2^_SNP=_0.051 (SE = 0.010)
Dark brown hair colour [42]	N_case_ = 146 394, N_control_ = 239 209, N = 385 603 (logistic)	UK Biobank (EUR)	https://atlas.ctglab.nl/traitDB/3498	h^2^_SNP=_0.124 (SE = 0.020)
Black hair colour [42]	N_case_ = 18 181, N_control_ = 367 422, N = 385 603 (logistic)	UK Biobank (EUR)	https://atlas.ctglab.nl/traitDB/3499	h^2^_SNP=_0.215 (SE = 0.040)
Tanning response [42]	N = 378 364 (linear) (1 = Get very tanned; 2 = Get moderately tanned; 3 = Get mildy or occasionally tanned; 4 = Never tan only burn)	UK Biobank (EUR)	https://atlas.ctglab.nl/traitDB/3274	h^2^_SNP=_0.079 (SE = 0.016)
Skin colour [42]	N = 381 433 (linear) (1 = Very fair; 2 = Fair; 3 = Light olive; 4 = Dark olive; 5 = Brown; 6 = Black)	UK Biobank (EUR)	https://atlas.ctglab.nl/traitDB/3273	h^2^_SNP_ = 0.074 (SE = 0.015)
Basal cell carcinoma (BCC) [42]	N_case_ = 12 890, N_control_ = 47 802, N = 60 692(logistic)	UK Biobank (EUR)	https://atlas.ctglab.nl/traitDB/3660	h^2^_SNP_ = 0.193 (SE = 0.026)
Squamous cell carcinoma (SCC) [43]	N_case_ = 7402, N_control_ = 286 892, N = 294 294 (logistic)	UK Biobank, QSkin Sun and Health Study, eMERGE, GERA (EUR)	https://www.ebi.ac.uk/gwas/studies/GCST90137412	h^2^_SNP_ = 0.579 (SE = 0.060)
Cutaneous malignant melanoma (CMM) [44]	N_case_ = 4898, N_control_ = 389 728, N = 394 626 (logistic)	UK Biobank (EUR)	https://www.ebi.ac.uk/gwas/studies/GCST90468141	h^2^_SNP_ = 0.060 (SE = 0.019)
Uveal melanoma (UM) [26]	N_case_ = 2426, N_control_ = 351 961, N = 354 387 (logistic)	Willis Eye Hospital, Penn Medicine Biobank (EUR)	https://www.ebi.ac.uk/gwas/studies/GCST90568461	h^2^_SNP_ = 0.004 (SE = 0.014)

**Table 2:** Number of shared significant SNPs (S_Het_  *P* < 5 × 10^−8^) with individual GWAS *P*-values <.001.

Cancer Trait	Pigmentation Trait	Shared Lead SNPs (*P* < 0.001)
**Squamous Cell Carcinoma (SCC)**		
**SCC**	Skin Colour	242
	Tanning Response	228
	Dark Brown Hair	207
	Blonde Hair	176
	Black Hair	160
	Light Brown Hair	119
	Red Hair	77
**Basal Cell Carcinoma (BCC)**		
**BCC**	Tanning Response	35
	Skin Colour	34
	Blonde Hair	32
	Dark Brown Hair	29
	Black Hair	26
	Light Brown Hair	19
	Red Hair	13
**Cutaneous Malignant Melanoma (CMM)**		
**CMM**	Tanning Response	14
	Blonde Hair	10
	Skin Colour	10
	Dark Brown Hair	9
	Black Hair	7
	Light Brown Hair	4
	Red Hair	4
**Uveal Melanoma (UM)**		
**UM**	Blonde Hair	4
	Tanning Response	3
	Dark Brown Hair	2
	Red Hair	1
	Skin Colour	1
	Black Hair	0
	Light Brown Hair	0

### SNP-based heritability and global genetic correlation

We estimated SNP-based heritability (h^2^_SNP_) and pairwise genetic correlations (rg) with LD score regression (LDSC) [45]. For case–control GWAS, heritability was converted to the liability scale using population prevalence from the Canadian Cancer Society (n.d.) and a 2019 hair-pigmentation review [46]; continuous traits are reported on the observed scale. LDSC applies standard quality control filters during munging (INFO > 0.9, MAF > 0.01, restriction to HapMap3 SNPs) and during the LD Score regression step removes SNPs with χ^2^ > max(80, N/1000), where N is the per-SNP sample size. All *P*-values were Bonferroni-corrected to assess robustness of our findings (*P*/55 individual tests).

### Multi-trait association analysis

We applied CPASSOC (Cross-Phenotype Association) R package (version 1.01) [47] to evaluate the extent of shared genetic architecture between seven pigmentation traits and four cancers. CPASSOC (http://hal.case.edu/zhu-web/) requires a correlation matrix to account for phenotypic correlations or overlapping samples across cohorts. This matrix was constructed using summary-level GWAS statistics from independent SNPs as recommended by Zhu *et al.* [47]. For datasets sourced from GWAS Catalogue and GWAS Atlas, where only summary-level data are accessible, LD patterns were taken from the 1000 Genomes Project (1KGP) via the PLINK2 Resources page (https://www.cog-genomics.org/plink/2.0/resources#phase3_1kg). Following the guidelines outlined in Li & Zhu [48], SNPs were pruned using PLINK2 with an LD threshold of r^2^ = 0.2 [49, 50] (https://www.cog-genomics.org/plink/2.0/), and variants with Z scores greater than ±1.96 were excluded when constructing the matrix to reduce bias.

CPASSOC generates two test statistics: S_Hom_ and S_Het_. S_Hom_ operates similarly to a fixed-effects meta-analysis whilst adjusting for cross-trait correlations [51]. S_Het_ extends S_Hom_ by accommodating heterogeneity in effect sizes across traits, and has been shown to provide improved power when genetic effects are heterogeneous across phenotypes [52]. As S_Het_ captures both homogeneous and heterogeneous pleiotropic signals, we report results obtained with S_Het_ throughout. CPASSOC utilizes a common set of SNPs present across all traits within each analysis, the number of common SNPs for each analysis are summarized in Supplementary Table S1.

Pleiotropy was tested (1) across all eleven traits (Table 1 and (2) pairwise between all cancer-pigmentation pairs. Lead SNPs from each multi-trait analysis were annotated with FUMA’s SNP2GENE (https://fuma.ctglab.nl/) [53].

After identifying genome-wide significant markers in the multi-trait CPASSOC analysis, we applied an additional secondary filter based on the *P*-values for the individual traits to minimize risk of false positives. An individual *P*-value filter of *P* < 0.001 was applied as our secondary threshold, representing a compromise in terms of true pleiotropic signals vs. false positives. We performed a sensitivity analysis across thresholds ranging from *P* < 0.05 to *P* < 5 × 10^−8^, evaluating the number of independent pleiotropic loci retaining nominal significance in at least one cancer and one pigmentation trait at each threshold (Supplementary Table S4). As another sensitivity analysis, we explored to which extent the LD-pruned significant markers for one pigmentary trait (in this case skin pigmentation) at different *P*-value thresholds (*P* < 0.001, *P* < 1 × 10^–5^ and *P* < 5 × 10^–8^) show inflated *P*-values for a cancer trait (in this case SCC), which would indicate evidence of pleiotropy. Inflation was substantial at all three thresholds and increased progressively at more stringent *P*-values (Supplementary Fig. S1).

### Cross-referencing uveal melanoma lead SNPs in eye colour GWAS

To investigate potential overlap between UM risk loci and eye colour variation, we cross-referenced the seven genome-wide SNPs reported in the UM GWAS by Mies *et al.* [26] with results from a recent GWAS of iris pigmentation conducted in CanPath cohort [28]. The CanPath sample size is 5641 (includes both males and females), with eye colour categorized as: 1 = blue and grey, 2 = green, 3 = hazel, and 4 = brown. We also conducted a pairwise analysis between eye colour and UM using CPASSOC.

### Post-association annotation

Lead SNPs and genomic risk loci were identified using FUMA SNP2GENE (v1.83) [53] with default parameters. Gene mapping was performed using positional mapping (±10 kb), expression quantitative trait locus (eQTL) mapping in eye tissue from EyeGEx [54], as well as skin sun-exposed and non-sun-exposed tissues from GTEx v8 [55, 56] (FDR < 0.05). Additional annotations for lead SNPs passing the dual threshold were obtained with SNPnexus (Version 5) (https://www.snp-nexus.org/v4/) [57–61]. Finally, colocalization data were retrieved from https://ngdc.cncb.ac.cn/colocdb/home [62] to determine whether associations observed between two traits could be attributed to a shared single variant, thus pointing to true pleiotropy, or to independent variants within the region.

**Figure 1:**
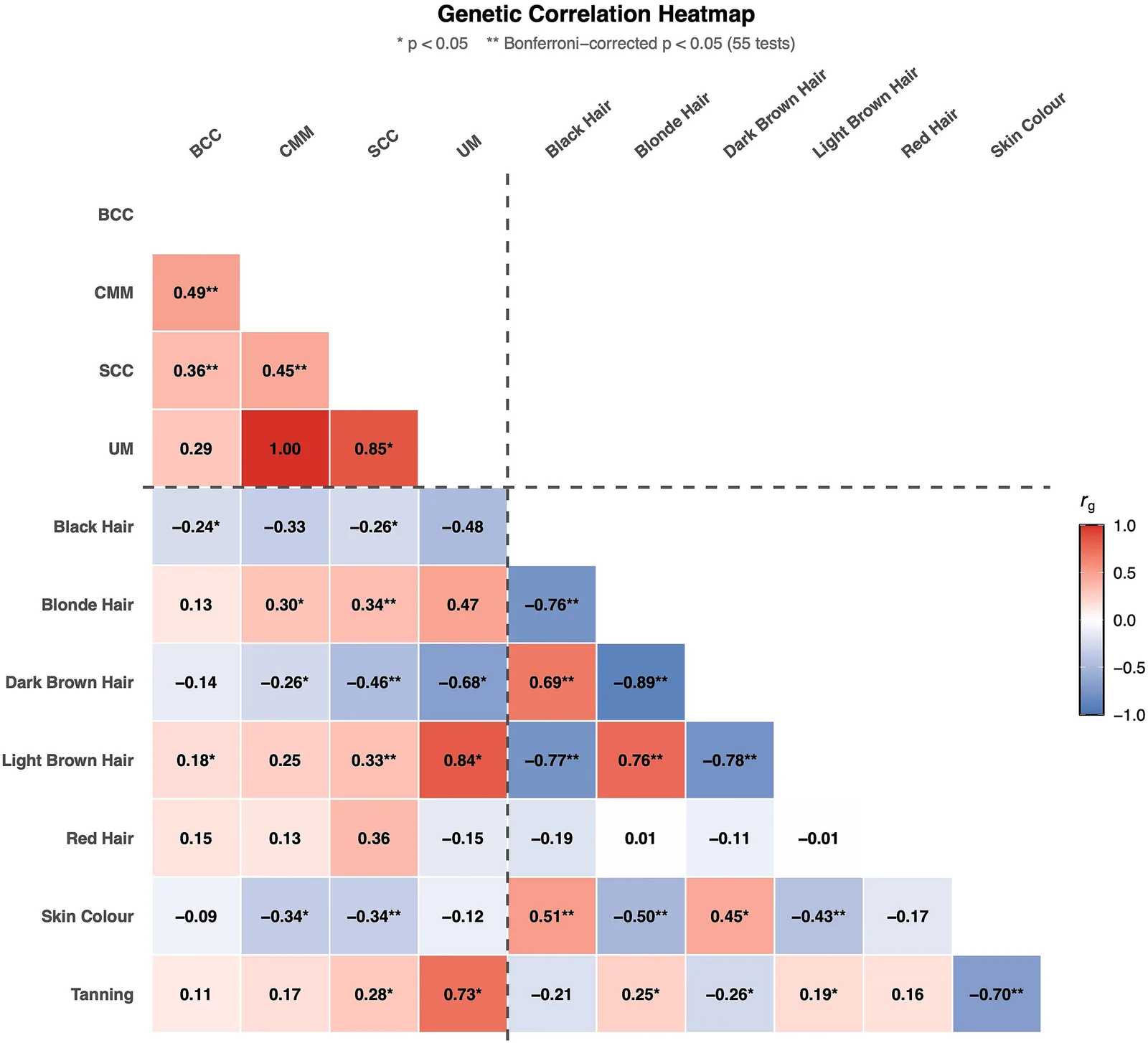
Heatmap of genetic correlations between cancer and pigmentation phenotypes. Nominally significant *P*-values (*P* < 0.05) are indicated with a single asterisk (^*^), *P*-values which survive Bonferroni multiple testing correction (*P*_Bonf_ < 0.05) are indicated with two asterisks (^**^).

## RESULTS

### Genome-wide datasets, SNP-based heritability, and global genetic correlation

Most traits exhibited measurable SNP-based heritability (h^2^_SNP_) (Table 1). The exceptions were eye colour and UM. The sample size for eye colour was very low, and LDSC was not able to generate heritability or correlation values. UM showed a very low heritability (h^2^_SNP_ = 0.004, SE = 0.014). The mean χ^2^ = 0.995 suggests limited polygenicity, and this may raise concerns about the liability of LDSC-based heritability estimates for this trait. The two traits with the lowest estimates of heritability, CMM (h^2^_SNP_ = 0.060, SE = 0.019) and UM, are characterized by relatively small number of cases, which likely reduced the GWAS statistical power. Although UM was retained in downstream analyses, results from LDSC genetic correlation analysis involving this trait should be interpreted with caution.

Genetic correlation results are presented in Fig. 1. Skin colour showed a significant negative correlation with SCC (rg = −0.337, *P* = 8.88 × 10^–4^, *p*_Bonf_ = 4.88 × 10^−2^) and a negative correlation in the same direction with CMM that did not survive Bonferroni correction (rg = −0.344, *P* = 2.59 × 10^−3^, *p*_Bonf_ = 0.142). Skin colour and tanning response were strongly negatively correlated (rg = −0.697, *P* = 4.73 × 10^–5^, *p*_Bonf_ = 2.60 × 10^−3^), reflecting their shared genetic architecture. Tanning response showed a nominally significant positive correlation with SCC (rg = 0.278, *P* = 4.11 × 10^–3^, *p*_Bonf_ = 0.226) and a positive but non-significant correlation with CMM (rg = 0.170, *P* = 0.119). Blonde hair showed a significant positive correlation with SCC that survived Bonferroni correction (rg = 0.344, *P* = 4.95 × 10^–6^, *p*_Bonf_ = 2.72 × 10^–4^), whilst red hair showed a positive but non-significant correlation (rg = 0.358, *P* = 0.069). The three skin cancers were also significantly positively correlated with each other: CMM-SCC (rg = 0.452, *P* = 1.21 × 10^–4^, *p*_Bonf_ = 6.64 × 10^–3^), BCC-CMM (rg = 0.487, *P* = 1.82 × 10^–4^, *p*_Bonf_ = 1.00 × 10^−2^), and BCC-SCC (rg = 0.357, *P* = 3.76 × 10^–6^, *p*_Bonf_ = 2.07 × 10^–4^) (Supplementary Table S2).

### Eleven-trait cancer-pigmentation association analysis (analysis 1)

Using S_Het_, CPASSOC identified 35 755 genome-wide significant markers. The Manhattan plot for the S_Het_ eleven-trait CPASSOC analysis is shown in Fig. 2 (Manhattan plot for the S_Hom_ eleven-trait CPASSOC analysis is shown is Supplementary Fig. S2). FUMA [53] highlighted 918 independent lead SNPs based on LD patterns (Supplementary Table S3), with several loci harbouring multiple independent signals. Loci were considered to be pleiotropic if they showed a CPASSOC multi-trait *P*-value < 5 × 10^–8^, and individual trait *P*-value < 0.001 for at least one cancer and one pigmentation trait). At this threshold, 303 of 918 independent loci (33.0%) showed nominal significance in at least one cancer and one pigmentation trait (Supplementary Table S3), with all four cancer types represented (SCC: 283 loci, 93.4%; BCC: 42 loci, 13.9%; CMM: 16 loci, 5.3%; UM: 7 loci, 2.3%) (Supplementary Table S4). The list of independent SNPs is available in Supplementary File S1.

**Figure 2:**
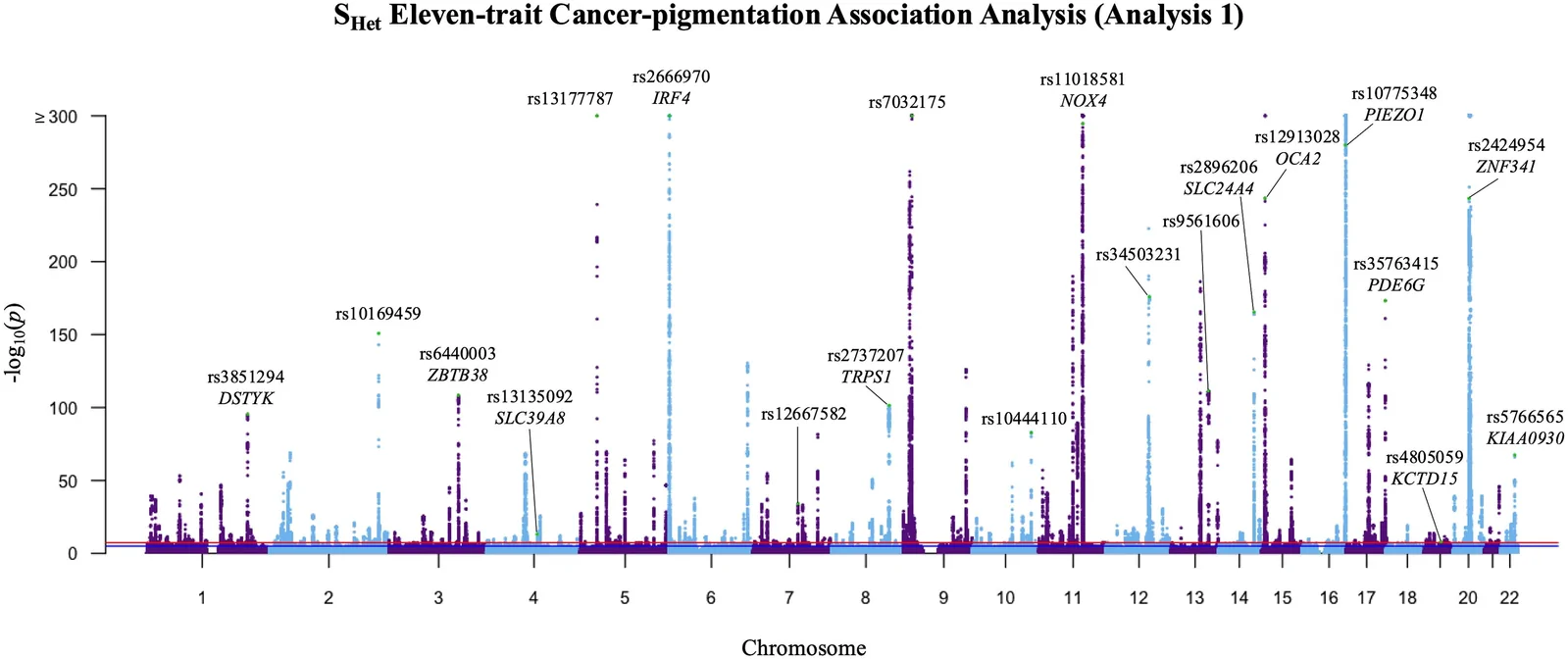
Manhattan plot depicting the results of the eleven-trait CPASSOC S_Het_ test. The red horizontal line represents the genome-wide significance threshold (*P* = 5 × 10^−5^), whilst the blue line indicates the suggestive significance threshold (*P* = 1 × 10^−5^); loci exceeding these thresholds are considered to show strong or potential associations, respectively. The y-axis has been truncated (ylim = 1.0 × 10^−300^) to show signals more clearly. Labelled SNPs are the top lead SNP per chromosome which have passed the dual threshold (S_Het_  *P*-value <5 × 10^−8^ and individual GWAS *P* < 0.001 in ≥1 cancer and ≥ 1 pigmentation trait)*.* In instances where SNPnexus [57–61] identified an overlapping gene, the gene name has been provided.

The strongest sharing was observed between SCC and skin colour (n = 242) and SCC and tanning response (n = 228), consistent with the established role of UV-sensitivity phenotypes in SCC risk. SCC demonstrated the greatest overall sharing across all seven pigmentation traits (n = 77–242), followed by BCC (n = 13–35) and CMM (n = 4–14). UM showed limited sharing with five of seven pigmentation traits (n = 1–4), with no shared lead SNPs observed for black hair or light brown hair at this threshold.

### Pairwise sharing (analysis 2)

After applying a secondary threshold of *P* < 0.001 to both traits, we identified SNPs shared between pigmentation traits and each skin cancer type: 773 with SCC, 188 with BCC, 131 with CMM, and 25 with UM. Sharing was broadest for SCC, which overlapped extensively with tanning response and skin colour, and to a lesser extent with the hair colour phenotypes. BCC overlap was led by skin colour, followed by the hair colour traits (dark brown, black, blonde, red, and light brown) and tanning response. CMM showed overlap distributed fairly evenly across all pigmentation traits, including hair colour, tanning response, and skin colour. UM showed the smallest degree of overlap, spread thinly across pigmentation traits and, uniquely amongst the four cancers, eye colour.

Genomic localization of the shared SNPs revealed a core set of pigmentation associated regions recurring across cancer types, most consistently *MC1R* (16q24.3), *ASIP* (20q11.22), *IRF4/EXOC2* (6p25.3), *TYR* (11q14.3), *GRM5* (11q14.2-q14.3), and *TPCN2* (11q13.3). Beyond this shared core, each cancer showed a distinct secondary profile. SCC signals were the most widely distributed, extending to *BNC2* (9p22.2-p22.3), *TYRP1* (9p23), *OCA2* (15q12-q13), *TRPM1* (15q13.3), *KITLG* (12q21.32), *PMEL* (12q13.2), *SLC24A4* (14q32.23), EDNRB (13q22.1–22.3), and *DCT* (13q32.1). BCC additionally implicated *OCA2/HERC2* (15q11.2/15q13) and *TRPM1* (15q13.3). CMM’s strongest signal was at *MC1R*, with secondary peaks at *OCA2/HERC2* alongside the shared core loci. UM was distinct in showing its strongest signal at *IRF4/EXOC2* rather than *MC1R*, with smaller contributions from *OCA2/HERC2, KITLG*, and *SLC45A2* (5p13.2).

When assessing direction of effect (Z scores based on betas and standard errors), most markers identified by CPASSOC followed expected trends: alleles associated with increased pigmentation and tanning response were protective against skin cancers, whereas alleles associated with blonde or red hair increased skin cancer risk. However, exceptions were observed, in particular for hair colour loci such as the *IRF4* region, which are discussed below. Overall, skin colour and tanning response emerged as the main drivers of genetic sharing with skin cancer risk. The summary of lead SNPs from the pigmentation-cancer runs are compiled in Supplementary File S1 and the relations of Z scores between pigmentary traits and skin cancers are provided in Supplementary Fig. S3.

CPASSOC S_Het_ analysis of UM and eye colour identified 318 genome-wide significant (based on S_Het_) SNPs, 12 of which FUMA [53] identified as lead SNPs (Supplementary Table S3). Only 2 passed the secondary *P*-value filter (*P* < 0.001 for both traits, Supplementary File S1). The Manhattan plot for this pairwise analysis is shown in Fig. 3.

**Figure 3:**
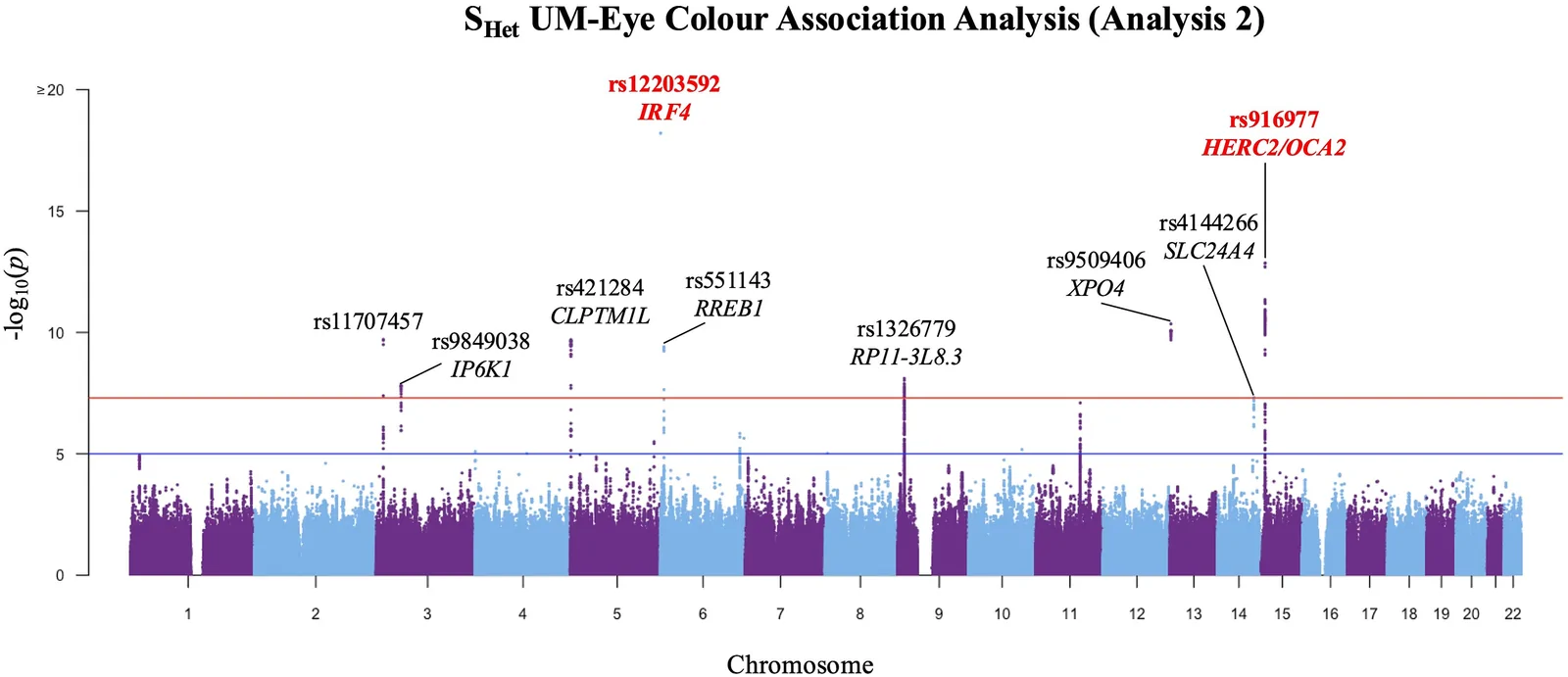
Manhattan plot depicting the results of the UM-eye colour CPASSOC S_Het_ test. The red horizontal line represents the genome-wide significance threshold (*P* = 5 × 10^−8^), whilst the blue line indicates the suggestive significance threshold (*P* = 1 × 10^–5^); loci exceeding these thresholds are considered to show strong or potential associations, respectively. The y-axis has been truncated (ylim = 1.0 × 10^−20^) to show signals more clearly. All lead SNPs identified by FUMA [53] are labelled; the SNPs in **red** have passed the dual threshold (S_Het_  *P*-value <5 × 10^−8^ and *P* < 0.001 in both the individual eye colour GWAS and uveal melanoma GWAS*.* In instances where SNPnexus [57–61] identified an overlapping gene, the gene name has been provided. The full plot (no truncated axis) can be found in Supplementary Fig. S4.

### Cross-referencing UM Lead SNPs in eye colour GWAS

Comparing UM GWAS lead SNPs [26] with a recent GWAS of iris colour [28], only two SNPs (rs12913832 and rs12203592) were found to be associated with eye colour. Effect directions showed that lighter iris colours significantly increased UM risk, consistent with previous studies which reported a connection between iris lightening alleles and risk of UM [27, 63]. Table 3 summarizes the effect sizes and *P*-values for the seven UM lead SNPs.

**Table 3:** Effect size (BETA) and *P*-values for seven lead SNPs associated with uveal melanoma [26] extracted from eye colour summary statistics [29].

rsID	CHR	BP	A1	A2	Gene	Beta (UM)	*P*-value (UM)	Beta (CanPath Eye Colour GWAS)	*P*-value (CanPath Eye Colour GWAS)
rs12913832	15	28365618	G	A	*HERC2*	0.3704	2.84E–15	−1.25795	0
rs12203592	6	396321	T	C	*IRF4*	0.441	7.11E–19	−0.166053	6.39E–09
rs421284	5	1325590	C	T	*CLPTMIL*	−0.2527	8.24E–11	0.000340512	0.987646
rs9849038	3	49771990	G	A	*IP6K1*	−0.2982	6.57E–09	0.0223195	0.443964
rs9509406	13	21440496	G	A	*XPO4*	−0.2578	1.90E–11	0.0305919	0.160688
rs11707457	3	14387519	C	T	*RP11-536I6.1*	−0.3641	8.57E–11	−0.0210785	0.546231
rs551143	6	7136772	G	A	*RREB1*	0.2964	1.84E–10	−0.0366971	0.1623

### Post-association annotation results

We explored the putative functional relevance of the 303 primary pleiotropic lead SNPs (CPASSOC SHet *P* < 5 × 10^−8^ and *P* < 0.001 in ≥1 cancer and ≥ 1 pigmentation trait) using SNPnexus [57–61] and FUMA SNP2GENE [53].

SNPnexus [57–61] annotations (Supplementary File S2) identified nonsynonymous variants in five genes: *CYP1B1, MLXIPL, DSTYK, SH2B3*, and *TPCN2*. Two SNPs were predicted deleterious by SIFT: rs3851294 at *DSTYK* (1q32.1) and rs34503231 at *SH2B3* (12q24.12). No variants were predicted damaging by PolyPhen2. Examining CADD scores across all 303 lead SNPs, 37 noncoding SNPs exceeded a PHRED score of 10. Amongst nonsynonymous variants, rs3851294 at *DSTYK* (CADD = 22.5) and rs34503231 at *SH2B3* (CADD = 20.3) exceeded a PHRED score of 20.

Using FUMA SNP2GENE [53], 1587 genes were identified as overlapping or located proximal to the primary pleiotropic lead SNPs (Supplementary File S3). eQTL mapping in skin tissues (GTEx skin sun-exposed lower leg and skin not sun-exposed suprapubic) revealed that 34 474 unique markers regulated 789 genes in skin. As expected, several genes overlapping or regulated by the pleiotropic SNPs play important roles in the pigmentary pathway, including *ASIP, BNC2, DCT, IRF4, KITLG, MC1R, OCA2, SLC24A4, SLC45A2, TPCN2, TYR,* and *TYRP1*, consistent with established genetic architecture of pigmentation traits and skin cancer susceptibility.

To complement our primary findings, we queried COLOCdb (https://ngdc.cncb.ac.cn/colocdb/home) [62] for the relevant pigmentary traits and skin cancers. Colocalization tests assess whether associations for different traits are driven by the same causal variant (true pleiotropy) or by distinct variants within a locus [64, 65]. For most markers, the data point to a single variant driving the signal for both traits (e.g. high posterior probabilities for the hypothesis of a single variant explaining the associations, or PP4) rather than multiple independent variants driving the signals (high PP3). Supplementary File S4 reports loci with strong evidence (PP4 ≥ 0.75 and PP4/PP3 ≥ 3) of sharing between pigmentary traits and CMM, SCC, or BCC. In line with our results, the strongest pleiotropy involved SCC and pigmentation, with additional support for BCC and CMM. Many regions from our multi-trait or pairwise analyses also colocalized, including *ATP11A, BNC2, GAS8, GCKR, HAL, HERC2/OCA2, IRF4, ITCH, LINC00886, LURAP1L, RXFP3, SLC24A4, SLC6A17, TPCN2, TRPS1*, and *TYRP1*. Beyond pigmentation, there was substantial sharing amongst the three skin cancers themselves, much of it seemingly driven by pigmentation-pathway loci.

In order to test the robustness of our results, we evaluated the concordance of *P*-values for markers with *P* < 0.001 in our skin cancer summary data with the *P*-values reported in a recent study of BCC, SCC, and CMM based on the VA Million Veteran Programme (MVP) biobank [66]. The European sample in this study included a larger number of cases for the three cancers than our summary data (~31 k for BCC, ~19 k for SCC, and ~ 45 k for CMM). Concordance was generally good but varied by trait, with the % of markers replicating at *P* < 0.001 and *P* < 0.05 in the MVP samples, respectively, as follows: BCC, 78.6%/92.9%; CMM, 56.25%/68.75%; SCC, 28.8%/48.4%.

These comparisons are reported in Supplementary File S5.

## DISCUSSION

This study employed multi-trait association approaches to examine shared genetic architecture between pigmentation traits (skin pigmentation, hair colour, and tanning response) and susceptibility to cutaneous and ocular cancers, with the novel inclusion of UM in a pleiotropy framework. Many genetic correlations remained significant after Bonferroni correction (Fig. 1). For example, SCC showed the expected negative correlation with skin colour and positive correlations with blonde hair and poor tanning response (Fig. 1). The same directional trends were observed for BCC and CMM, though fewer correlations survived Bonferroni correction, likely reflecting reduced statistical power due to smaller case numbers. These results align with epidemiological patterns linking lighter pigmentation and burning tendency to increased skin cancer risk, and conversely, darker pigmentation and greater tanning ability to decreased risk [10, 67]. Significant correlations between CMM, SCC, BCC, and UM indicate overlapping susceptibility. It should be noted that CMM and UM showed low heritability, likely reflecting limited GWAS power due to small case numbers (<5000 for CMM; <2500 for UMM). Consistent with this, several variants that were genome-wide significant in multi-trait tests showed only nominal signals for CMM (*P* < 0.05) despite strong effects in pigmentation traits, suggesting larger CMM and UM GWAS will reveal additional shared pleiotropy.

CPASSOC multi-trait and pairwise analyses reinforced substantial pleiotropy between pigmentary traits and skin cancers. Well-known pigmentation loci, including *MC1R, ASIP, TYR, BNC2*, and *IRF4*, were hubs of shared risk, in agreement with many previous studies [12, 43, 67–70], with additional signals in other melanin-related genes, such as *OCA2, SLC24A4, DCT,* or *TYRP1*. CPASSOC also identified several regions that did not reach genome-wide significant in individual GWAS but became significant in the multi-trait analysis, or the pairwise analyses (Supplementary File S1). This highlights the value of multi-trait approaches for detecting shared loci and suggests larger GWAS will reveal further pleiotropy.

The 11-trait CPASSOC analysis highlights the highly pleiotropic architecture of pigmentary traits. Markers showing genome-wide significance typically had *P* < 0.001 across 2–7 pigmentary traits, consistent with shared pigmentation-pathway genes that in turn influence cancer risk through melanin’s protective effect. The direction of effects of alleles identified in the CPASSOC meta-analysis generally aligned with epidemiological evidence: lighter skin, hair and iris pigmentation and poor tanning response increased risk for all the cancers analyzed. Notably, the *IRF4* region was an exception; whilst most pigmentation loci link lighter skin and poor tanning with lighter iris and hair colour, *IRF4* variants associate instead with light skin, poor tanning, and blue eyes but paradoxically with dark hair [71, 72]. In our analysis, the *IRF4* rs12203592 T allele followed this unusual pattern yet still conferred increased BCC and UM risk, despite its association with darker hair. This reinforces the idea that skin colour and tanning response are the primary drivers of skin cancer susceptibility. This is consistent with our findings that the strongest correlations of effect sizes (Z-scores) between pigmentation traits and skin cancer are observed for skin colour and tanning response, with the alleles showing the strongest effects on the pigmentary traits also resulting in the highest increases in skin cancer risk (Supplementary Fig. S3).

This study also breaks new ground by including UM within a pleiotropy framework. Although overlap with pigmentation traits was limited, likely reflecting low statistical power of the available UM GWAS, evidence of pleiotropy emerged from our multi-trait analysis and the follow-up of lead UM-associated SNPs in an eye colour GWAS (Table 3). Notably, loci near *IRF4* and *HERC2*, regions strongly associated with blue eye colour, also appear to play a role in UM susceptibility. As larger UM GWAS efforts become available, additional shared loci between UM and pigmentary traits will likely be uncovered.

We acknowledge that this study has several limitations. First, the number of cancer cases included in the GWAS studies analyzed here is quite low (~13 000 for BCC and < 2500 for UM), limiting these GWAS to markers with relatively strong effects. Second, the number of markers available varied across analyses (~3.8 to 9 million; Supplementary Table S1), affecting the resolution of our CPASSOC results, which require markers common to all analyzed traits. Third, our study focused on populations of European ancestry; GWAS for skin cancer and pigmentary traits exist in other population groups, but with more limited sample sizes and, given the lower incidence of skin cancer outside European ancestry populations [73], reduced statistical power (e.g. Verma *et al.* [66] reported only ~200–800 SCC, CMM, and BCC cases in African American, African Caribbean, and Hispanic/Latino GWAS). Finally, the genetic architecture of pigmentary traits is known to differ between population groups, reflecting at least partly independent selective histories in Europeans and East Asians [74, 75]; extending future analyses to East Asian and other high-latitude populations would help test the generalizability of our findings.

## CONCLUSION AND IMPLICATIONS: SKIN CANCER RISK AND EVOLUTIONARY MEDICINE

Our results reinforce that light pigmentation and poor tanning response increase skin cancer risk, involving many well-documented pigmentation genes (*MC1R, TYR, TYRP1, OCA2*, and *ASIP* amongst others) [10, 67]. This reflects evolutionary processes as modern humans moved into high-latitude environments, where depigmentation was positively selected to support vitamin D synthesis under lower, more seasonal UV radiation [10, 67]. Ancient DNA studies show that allele frequency changes for light pigmentation mainly occurred from the Neolithic to Bronze Age, likely reflecting dietary shifts towards poorer vitamin D sources [76]; pigmentation variants under selection in these studies, including rs16891982 (*SLC45A2*), rs1126809 (*TYR*), and rs12913832 (*HERC2*), appear prominently in our results [4, 77]. More generally, our analyses identified pleiotropy in regions harbouring genes previously identified in selection scans, including *SLC45A2, TYR, OCA2/HERC2*, and *ASIP*, amongst others [78]. Variants that reduced melanin content to support high-latitude adaptation are now linked to increased skin cancer risk—a striking example of evolutionary mismatch. Epidemiological data indicate that skin cancer incidence has risen in recent decades [79], underscoring the need for further work to uncover the genetic architecture of these diseases.

## Supplementary Material

Supplementary_Tables_Figures_Aug8_eoag021

## Data Availability

All data used in this study is publicly available (GWAS summary statistics) and links for the sites where the summary statistics were downloaded have all been provided.
