# Supplementary material for: Multi-trait analysis of pigmentation traits in relation to skin and ocular cancers: Relevance for evolutionary medicine
Source: Evol Med Public Health. 2026 Sep 10;14(1):1–13. doi: 10.1093/emph/eoag021 (PMC13656177; doi:10.1093/emph/eoag021)
Supplement: Supplementary file 1 [file supplementary_tables_figures_aug8_eoag021.docx]

**Supplementary Material**

**Supplementary Tables.**

**Table S1.** **Number of single nucleotide polymorphisms (SNPs) included in each CPASSOC analysis.** SNPs represent the intersection of markers present across all summary statistics files within each analysis, following harmonization to the UKB reference panel (hg37). BCC, basal cell carcinoma; CMM, cutaneous malignant melanoma; SCC, squamous cell carcinoma; UM, uveal melanoma.

| **Analysis 1** | |
| --- | --- |
| 11-Trait | 3,768,363 |
| **Analysis 2** | |
| BCC-Pigmentation | 9,066,843 |
| CMM-Pigmentation | 8,966,247 |
| SCC-Pigmentation | 5,124,175 |
| UM-Pigmentation | 5,115,410 |
| **Analysis 3** | |
| BCC-Black hair | 9,066,843 |
| BCC-Blonde hair | 9,066,843 |
| BCC-Dark brown hair | 9,066,843 |
| BCC-Light brown hair | 9,066,843 |
| BCC- Red hair | 9,066,835 |
| BCC-Skin colour | 9,066,843 |
| BCC-Tanning | 9,066,843 |
| CMM-Black hair | 8,966,257 |
| CMM-Blonde hair | 8,966,257 |
| CMM-Dark brown hair | 8,966,257 |
| CMM-Light brown hair | 8,966,257 |
| CMM- Red hair | 8,966,247 |
| CMM-Skin colour | 8,966,257 |
| CMM-Tanning | 8,966,257 |
| SCC-Black hair | 5,124,175 |
| SCC-Blonde hair | 5,124,175 |
| SCC-Dark brown hair | 5,124,175 |
| SCC-Light brown hair | 5,124,175 |
| SCC- Red hair | 5,124,175 |
| SCC-Skin colour | 5,124,175 |
| SCC-Tanning | 5,124,175 |
| UM-Black hair | 5,115,410 |
| UM-Blonde hair | 5,115,410 |
| UM-Dark brown hair | 5,115,410 |
| UM-Light brown hair | 5,115,410 |
| UM- Red hair | 5,115,410 |
| UM-Skin colour | 5,115,410 |
| UM-Tanning | 5,115,410 |
| UM-Eye colour | 3,618,283 |

**Table S2. Genetic correlations (rg) calculated using LDSC (Bulik-Sullivan et al., 2015).** *p*-values less than 0.05 are highlighted in purple, Bonferroni corrected *p*-values less than 0.05 are highlighted in green.

| **Trait1** | **Trait2** | **rg** | **SE** | **Z** | **P** | **P_Bonf** |
| --- | --- | --- | --- | --- | --- | --- |
| BCC | SCC | 0.3568 | 0.0771 | 4.624 | 3.76E-06 | 0.000207 |
| BCC | CMM | 0.4869 | 0.13 | 3.7429 | 0.0001819 | 0.01001 |
| BCC | BlackHair | -0.236 | 0.0953 | -2.4742 | 0.01335 | 0.7344 |
| BCC | LightBrownHair | 0.1792 | 0.0844 | 2.119 | 0.03409 | 1 |
| BCC | BlondeHair | 0.1288 | 0.0736 | 1.7514 | 0.07987 | 1 |
| BCC | DarkBrownHair | -0.1378 | 0.0813 | -1.6984 | 0.08943 | 1 |
| BCC | Tanning | 0.1091 | 0.0842 | 1.2981 | 0.1943 | 1 |
| BCC | SkinColour | -0.0857 | 0.0828 | -1.0404 | 0.2982 | 1 |
| BCC | RedHair | 0.1476 | 0.1763 | 0.8357 | 0.4033 | 1 |
| BCC | UM | 0.2914 | 0.4091 | 0.7156 | 0.4742 | 1 |
| BlackHair | BlondeHair | -0.7587 | 0.1353 | -5.6064 | 2.07E-08 | 1.14E-06 |
| BlackHair | DarkBrownHair | 0.6887 | 0.1378 | 4.9956 | 5.87E-07 | 3.23E-05 |
| BlackHair | LightBrownHair | -0.766 | 0.1765 | -4.3405 | 1.42E-05 | 0.0007817 |
| BlackHair | SkinColour | 0.5118 | 0.1206 | 4.2434 | 2.20E-05 | 0.001211 |
| BlackHair | SCC | -0.2567 | 0.0797 | -3.2206 | 0.001279 | 0.07035 |
| BlackHair | Tanning | -0.2095 | 0.1083 | -1.9362 | 0.05285 | 1 |
| BlackHair | CMM | -0.3349 | 0.1736 | -1.9289 | 0.05374 | 1 |
| BlackHair | UM | -0.4758 | 0.4309 | -1.1074 | 0.2681 | 1 |
| BlackHair | RedHair | -0.192 | 0.2396 | -0.8 | 0.4237 | 1 |
| BlondeHair | DarkBrownHair | -0.892 | 0.1343 | -6.6409 | 3.12E-11 | 1.71E-09 |
| BlondeHair | LightBrownHair | 0.7604 | 0.1268 | 6 | 1.97E-09 | 1.09E-07 |
| BlondeHair | SkinColour | -0.5019 | 0.1055 | -4.758 | 1.96E-06 | 0.0001076 |
| BlondeHair | SCC | 0.3443 | 0.0754 | 4.5669 | 4.95E-06 | 0.0002723 |
| BlondeHair | Tanning | 0.2457 | 0.0897 | 2.7391 | 0.00616 | 0.3388 |
| BlondeHair | CMM | 0.3047 | 0.1298 | 2.3448 | 0.01904 | 1 |
| BlondeHair | UM | 0.4743 | 0.3316 | 1.4273 | 0.1535 | 1 |
| BlondeHair | RedHair | 0.008 | 0.1775 | 0.0449 | 0.9642 | 1 |
| CMM | SCC | 0.4517 | 0.1175 | 3.8447 | 0.0001207 | 0.006637 |
| CMM | SkinColour | -0.3441 | 0.1141 | -3.0132 | 0.002585 | 0.1422 |
| CMM | DarkBrownHair | -0.2612 | 0.1286 | -2.036 | 0.04175 | 1 |
| CMM | UM | 1 | 0.8014 | 1.9412 | 0.05224 | 1 |
| CMM | LightBrownHair | 0.2546 | 0.1353 | 1.88 | 0.06011 | 1 |
| CMM | Tanning | 0.1702 | 0.109 | 1.56 | 0.1188 | 1 |
| CMM | RedHair | 0.1329 | 0.2555 | 0.5174 | 0.6048 | 1 |
| DarkBrownHair | LightBrownHair | -0.7773 | 0.1404 | -5.5357 | 3.10E-08 | 1.71E-06 |
| DarkBrownHair | SCC | -0.4578 | 0.0951 | -4.8157 | 1.47E-06 | 8.07E-05 |
| DarkBrownHair | SkinColour | 0.4477 | 0.1377 | 3.25 | 0.001154 | 0.06347 |
| DarkBrownHair | Tanning | -0.2597 | 0.1111 | -2.3364 | 0.01947 | 1 |
| DarkBrownHair | UM | -0.6784 | 0.3323 | -2.0423 | 0.04113 | 1 |
| DarkBrownHair | RedHair | -0.1108 | 0.3056 | -0.3615 | 0.7177 | 1 |
| LightBrownHair | SCC | 0.3282 | 0.0761 | 4.313 | 1.61E-05 | 0.0008859 |
| LightBrownHair | SkinColour | -0.4262 | 0.1137 | -3.7429 | 0.0001819 | 0.01001 |
| LightBrownHair | UM | 0.8395 | 0.4009 | 2.0909 | 0.03654 | 1 |
| LightBrownHair | Tanning | 0.1853 | 0.0928 | 2 | 0.0455 | 1 |
| LightBrownHair | RedHair | -0.014 | 0.2138 | -0.0677 | 0.946 | 1 |
| RedHair | SCC | 0.358 | 0.197 | 1.818 | 0.06907 | 1 |
| RedHair | Tanning | 0.1575 | 0.2745 | 0.5755 | 0.565 | 1 |
| RedHair | SkinColour | -0.1676 | 0.3023 | -0.5531 | 0.5802 | 1 |
| RedHair | UM | -0.1503 | 0.5522 | -0.2717 | 0.7858 | 1 |
| SCC | SkinColour | -0.3371 | 0.1014 | -3.3238 | 0.000888 | 0.04884 |
| SCC | Tanning | 0.278 | 0.0968 | 2.8696 | 0.00411 | 0.2261 |
| SCC | UM | 0.8531 | 0.336 | 2.5355 | 0.01123 | 0.6176 |
| SkinColour | Tanning | -0.6967 | 0.1714 | -4.0687 | 4.73E-05 | 0.0026 |
| SkinColour | UM | -0.1161 | 0.2911 | -0.3958 | 0.6922 | 1 |
| Tanning | UM | 0.7315 | 0.3522 | 2.0833 | 0.03722 | 1 |

**Table S3. Independent lead SNPs (as defined by FUMA) from CPASSOC S_Het_ tests (Zhu et al., 2015).** Excel file contains the following sheets:

| 11Trait_all | N/A | N/A | all | 918 |
| --- | --- | --- | --- | --- |
| 11Trait_0.001 | N/A | N/A | filtered | 303 |
| BCC_Black_all | BCC | BlackHair | all | 440 |
| BCC_Black_0.001 | BCC | BlackHair | filtered | 47 |
| BCC_Blonde_all | BCC | BlondeHair | all | 775 |
| BCC_Blonde_0.001 | BCC | BlondeHair | filtered | 47 |
| BCC_DkBrown_all | BCC | DarkBrown | all | 742 |
| BCC_DkBrown_0.001 | BCC | DarkBrown | filtered | 49 |
| BCC_LtBrown_all | BCC | LightBrown | all | 263 |
| BCC_LtBrown_0.001 | BCC | LightBrown | filtered | 32 |
| BCC_Red_all | BCC | RedHair | all | 294 |
| BCC_Red_0.001 | BCC | RedHair | filtered | 36 |
| BCC_Skin_all | BCC | SkinColour | all | 562 |
| BCC_Skin_0.001 | BCC | SkinColour | filtered | 53 |
| BCC_Tan_all | BCC | Tanning | all | 928 |
| BCC_Tan_0.001 | BCC | Tanning | filtered | 49 |
| CMM_Black_all | CMM | BlackHair | all | 365 |
| CMM_Black_0.001 | CMM | BlackHair | filtered | 32 |
| CMM_Blonde_all | CMM | BlondeHair | all | 580 |
| CMM_Blonde_0.001 | CMM | BlondeHair | filtered | 30 |
| CMM_DkBrown_all | CMM | DarkBrown | all | 677 |
| CMM_DkBrown_0.001 | CMM | DarkBrown | filtered | 35 |
| CMM_LtBrown_all | CMM | LightBrown | all | 208 |
| CMM_LtBrown_0.001 | CMM | LightBrown | filtered | 16 |
| CMM_Red_all | CMM | RedHair | all | 235 |
| CMM_Red_0.001 | CMM | RedHair | filtered | 28 |
| CMM_Skin_all | CMM | SkinColour | all | 555 |
| CMM_Skin_0.001 | CMM | SkinColour | filtered | 30 |
| CMM_Tan_all | CMM | Tanning | all | 753 |
| CMM_Tan_0.001 | CMM | Tanning | filtered | 35 |
| SCC_Black_all | SCC | BlackHair | all | 421 |
| SCC_Black_0.001 | SCC | BlackHair | filtered | 194 |
| SCC_Blonde_all | SCC | BlondeHair | all | 703 |
| SCC_Blonde_0.001 | SCC | BlondeHair | filtered | 231 |
| SCC_DkBrown_all | SCC | DarkBrown | all | 694 |
| SCC_DkBrown_0.001 | SCC | DarkBrown | filtered | 256 |
| SCC_LtBrown_all | SCC | LightBrown | all | 330 |
| SCC_LtBrown_0.001 | SCC | LightBrown | filtered | 143 |
| SCC_Red_all | SCC | RedHair | all | 220 |
| SCC_Red_0.001 | SCC | RedHair | filtered | 90 |
| SCC_Skin_all | SCC | SkinColour | all | 489 |
| SCC_Skin_0.001 | SCC | SkinColour | filtered | 286 |
| SCC_Tan_all | SCC | Tanning | all | 715 |
| SCC_Tan_0.001 | SCC | Tanning | filtered | 308 |
| UM_Black_all | UM | BlackHair | all | 292 |
| UM_Black_0.001 | UM | BlackHair | filtered | 5 |
| UM_Blonde_all | UM | BlondeHair | all | 468 |
| UM_Blonde_0.001 | UM | BlondeHair | filtered | 7 |
| UM_DkBrown_all | UM | DarkBrown | all | 517 |
| UM_DkBrown_0.001 | UM | DarkBrown | filtered | 5 |
| UM_Eye_all | UM | EyeColour | all | 12 |
| UM_Eye_0.001 | UM | EyeColour | filtered | 2 |
| UM_LtBrown_all | UM | LightBrown | all | 140 |
| UM_LtBrown_0.001 | UM | LightBrown | filtered | 2 |
| UM_Red_all | UM | RedHair | all | 156 |
| UM_Red_0.001 | UM | RedHair | filtered | 4 |
| UM_Skin_all | UM | SkinColour | all | 394 |
| UM_Skin_0.001 | UM | SkinColour | filtered | 6 |
| UM_Tan_all | UM | Tanning | all | 525 |
| UM_Tan_0.001 | UM | Tanning | filtered | 6 |

**Table S4**. Sensitivity analysis for the secondary individual trait *p*-value threshold applied to 11-trait CPASSOC results. Cancer and pigmentation (C+P) loci, independent lead SNPs (identified by FUMA SNP2GENE) with secondary *p* < threshold in ≥1 cancer trait and ≥1 pigmentation trait. BCC, basal cell carcinoma; CMM, cutaneous malignant melanoma; SCC, squamous cell carcinoma; UM, uveal melanoma.

| **Secondary Threshold** | **C+P Loci** | **BCC** | **CMM** | **SCC** | **UM** | **% of 918 Lead SNPs** |
| --- | --- | --- | --- | --- | --- | --- |
| p < 0.05 | 603 | 186 | 126 | 496 | 67 | 65.7% |
| p < 0.01 | 435 | 92 | 44 | 383 | 26 | 47.4% |
| p < 0.001 | 303 | 42 | 16 | 283 | 7 | 33.0% |
| p < 0.0001 | 201 | 22 | 8 | 192 | 1 | 21.9% |
| p < 1×10^-5^ | 150 | 14 | 1 | 144 | 0 | 16.3% |
| p < 5×10^-8^ | 89 | 3 | 1 | 86 | 0 | 9.7% |

**Supplementary Figures**.

**Figure S1.** QQ plots showing inflation of p-values for SCC of LD-pruned significant markers for skin pigmentation at three pvalue-thresholds (Figure A:p<0.001, Figure B:p<1x10-5 and Figure C:p<5x10-8).

**A)**

**B)**

**C)**

**Figure S2. Manhattan plot depicting the results of the eleven-trait CPASSOC S_Hom_ test.** The red horizontal line represents the genome-wide significance threshold (*p=*5×10^-5^), while the blue line indicates the suggestive significance threshold (*p=*1×10^-5^); loci exceeding these thresholds are considered to show strong or potential associations, respectively.

**Figure S3.** **Scatterplots of Z scores of pigmentary traits and skin cancers.**

**A)**

**B)**

**C)**

**D)**

**E)**

**F)**

**G)**

**H)**

**I)**

**J)**

**K)**

**L)**

**M)**

**N)**

**O)**

**P)**

**Q)**

**R)**

**S)**

**T)**

**U)**

**V)**

**W)**

**X)**

**Y)**

**Z)**

**AA)**

**AB)**

**AC)**

**Figure S4. Manhattan plot depicting the results of the UM-eye colour CPASSOC S_Het_ test.** The red horizontal line represents the genome-wide significance threshold (*p=*5×10^-8^), while the blue line indicates the suggestive significance threshold (*p=*1×10-5); loci exceeding these thresholds are considered to show strong or potential associations, respectively. Lead SNPs are labelled, and in red are SNPs which have passed the dual threshold. threshold (S_Het_ *p*-value <5×10^-8^ and *p*<0.001 in both the individual eye colour GWAS and uveal melanoma GWAS*.*

**Supplementary Files**

Supplementary files S1 to S5 are available in the following figshare link:

<https://doi.org/10.6084/m9.figshare.32301924>

**Supplementary Material References**

Bulik-Sullivan, B., Finucane, H. K., Anttila, V., Gusev, A., Day, F. R., Loh, P.-R., ReproGen Consortium, Psychiatric Genomics Consortium, Genetic Consortium for Anorexia Nervosa of the Wellcome Trust Case Control Consortium 3, Duncan, L., Perry, J. R. B., Patterson, N., Robinson, E. B., Daly, M. J., Price, A. L., & Neale, B. M. (2015). An atlas of genetic correlations across human diseases and traits. *Nature Genetics*, *47*(11), 1236–1241. <https://doi.org/10.1038/ng.3406>

Zhu, X., Feng, T., Tayo, B. O., Liang, J., Young, J. H., Franceschini, N., Smith, J. A., Yanek, L. R., Sun, Y. V., Edwards, T. L., Chen, W., Nalls, M., Fox, E., Sale, M., Bottinger, E., Rotimi, C., COGENT BP Consortium, Liu, Y., McKnight, B., … Redline, S. (2015). Meta-analysis of correlated traits via summary statistics from GWASs with an application in hypertension. *The American Journal of Human Genetics*, *96*(1), 21–36. https://doi.org/10.1016/j.ajhg.2014.11.011
